# Stafne Bone Defect: Report of Two Cases

**DOI:** 10.1155/2012/654839

**Published:** 2012-09-23

**Authors:** A. P. Münevveroğlu, K. C. Aydın

**Affiliations:** ^1^Pedodonti Ana Bilim Dalı, Dişhekimliği Fakültesi, İstanbul Medipol Üniversitesi, 34083 Fatih, Turkey; ^2^Diş ve Çene Radyolojisi Anabilim Dalı, Dişhekimliği Fakültesi Ağız, İstanbul Medipol Üniversitesi, 34083 Fatih, Turkey

## Abstract

Stafne bone defects are asymptomatic lingual bone depressions of the lower jaw. In 1942, Stafne described for the first time 35 asymptomatic, radiolucent cavities, unilaterally located in the posterior region of the mandible, between the mandibular angle and the third molar, below the inferior dental canal and slightly above the basis mandibulae. In this study, the clinical and radiological characteristics of 2 cases of Stafne bone defects were described. Orthopantomograph and CBCT were used for diagnosing the defects. The bone defects of two patients in this study were asymptomatic and any other bone lesions, such as cysts and tumors, were excluded because no signs of inflammatory or tumoral changes were evident Therefore, surgery was not considered and the patients were followed for 1 year. Stafne bone defect was an incidental finding, presenting no evolutionary changes, and as such conservatory therapy based on periodic controls was indicated. Currently, complementary techniques such as CT are sufficient to establish a certain diagnosis.

## 1. Introduction

In 1942, Stafne [1] described for the first time 35 asymptomatic, radiolucent cavities, unilaterally located in the posterior region of the mandible, between the mandibular angle and the third molar, below the inferior dental canal and slightly above the basis mandibulae [1–6]. Radiographically, the cortical outline of the bone defect is denser and thicker than that of odontogenic cysts [2].

The exact pathogenesis is still obscure. Stafne suggested that the cavity could result from a failure of normal bone deposition in the region formerly occupied by cartilage [1, 5, 7]. However, the most widely accepted view is that the cavities develop as a result of a localized pressure atrophy of the lingual surface of the mandible from the adjacent salivary gland [5, 8]. Due to the multiple explanations offered as to its etiopathogenesis, this entity has been given numerous names, particularly, static bone cyst, lingual mandibular bone defect, Stafne bone cavity, idiopathic bone cavity, and lingual mandibular bone depression [8]. The shape of a lingual bone cavity can be round or oval, and it varies from 1 to 3 cm in diameter [2].

Bone defects of Stafne frequently affect men in their fifth or seventh decade of life and show a prevalence between 0.10% and 0.48% [3, 9]. They usually contain ectopic salivary gland tissue and do not require surgery [3, 4, 10].

In addition to occurring more commonly in the posterior region of the mandible (posterior variant), Stafne bone defect may also appear in the anterior region (anterior variant) and in the ascending ramus of the mandible (mandibular ramus variant) [11]. Posterior Stafne bone defects can be readily diagnosed because of their unique location in the radiographic examination; however, anterior bone defects may be misdiagnosed and confused with several pathologic entities, such as traumatic and cystic lesions or tumors of the jaw [3]. Normally, it is unilateral, with some reports of bilateral occurrence [3–6]. Double unilateral occurrence is rare, and bilocular occurrence is even rarer [3].

The objective of this study is to describe the clinical and radiological characteristics of 2 cases of Stafne bone defect.

**Case 1**

**Figure 1:**
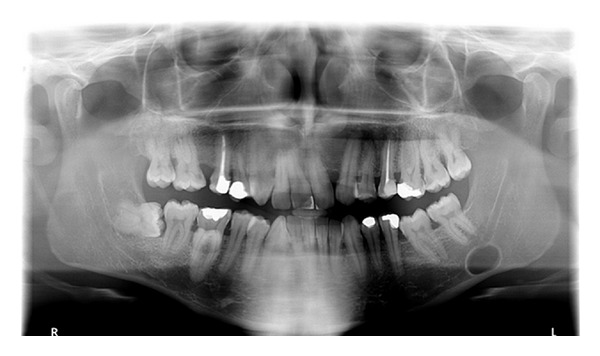
Panoramic radiography showing the radiolucent area.

**Figure 2:**
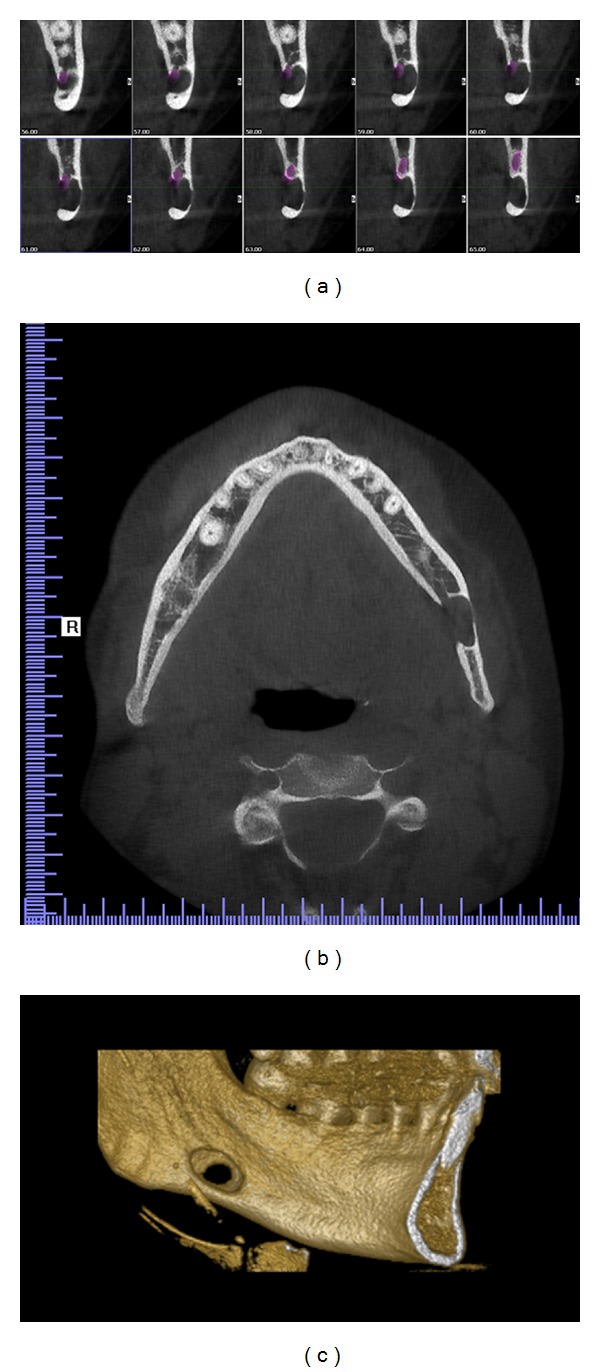
CBCT images: (a) sagital view displaying continuous 1 mm width sections, (b) horizontal view displaying the cavity outline with diminished buccal cortical bone, and (c) 3D reconstruction of the left mandible displaying buccal cortical bone reduction.

A 31-year-old asymptomatic male patient was referred to Istanbul Medipol University, Faculty of Dentistry, Department of Dentomaxillofacial Radiology in order to undergo routine panoramic radiography. The patient was misdiagnosed in behalf of a cystic lesion of his left mandible by a former dentist. Medical history and dental history were not contributory. Palpation of the defect was not painful, and the cavity could be palpated by bidigital palpation. Cone beam computerized tomography (CBCT) was found appropriate for further evaluation (see Figures 2 and 4). Results showed an oval-shaped, radiolucent area of cystic aspect and regular, well-defined cortical outline with a little buccal cortical resorpton. Its longest axis was placed horizontally in the left hemimandible. This lithic area, located under the lower left second molar, was anterior to the mandibular angle. The lower wall of the mandibular canal which was visible within the radiolucent area showed that there could be a neighboring relationship, but not an involvement, of the inferior alveolar nerve (Figure 1). Patient displayed no pain or paresthesia. Lingual wall of the basal bone displayed involvement of the lesion and dimensions of the defect 14 × 10 × 6.4 mm depth (mesiodistal length, inferosuperior height, buccolingual depth).

A diagnosis of idiopathic bone defect was made and it was decided that the patient would undergo a 6-month follow-up period.

**Case 2**

**Figure 3:**
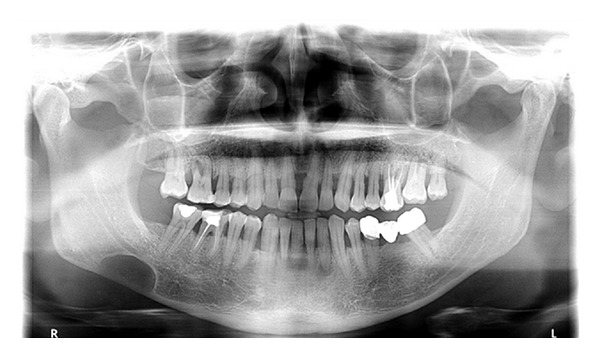
Panoramic radiography showing the radiolucent area.

**Figure 4:**
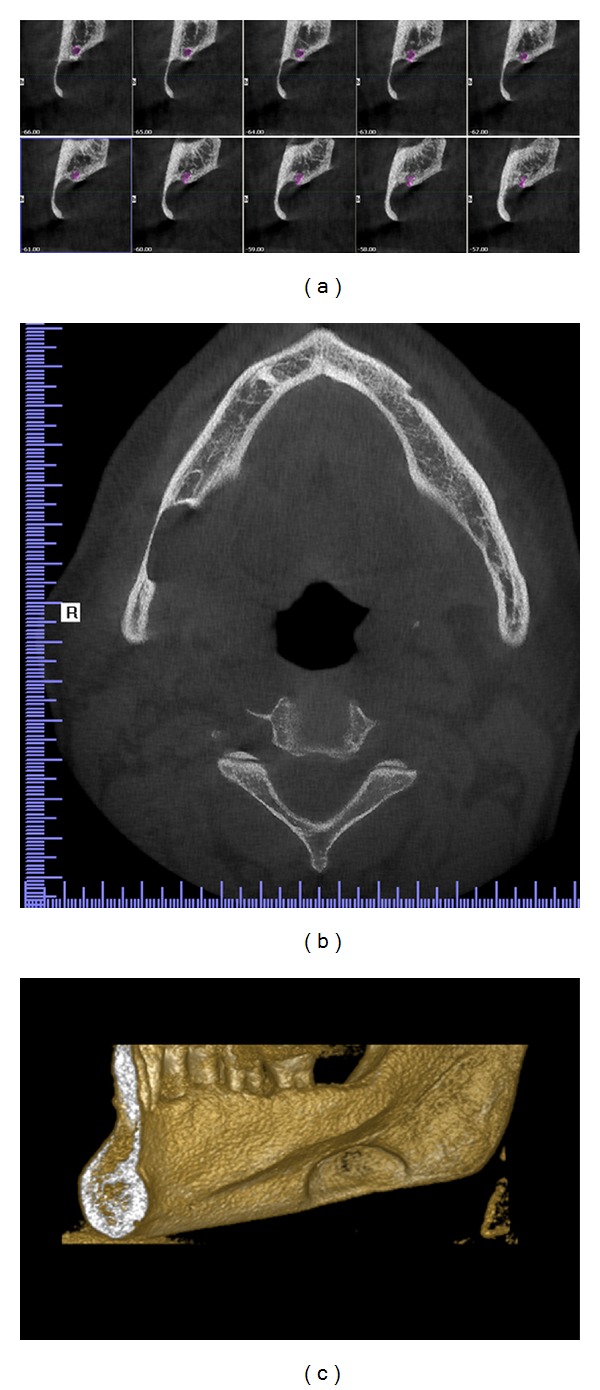
CBCT images: (a) sagital view displaying continuous 1 mm width sections, (b) horizontal view displaying the cavity outline, and (c) 3D reconstruction of the right mandible displaying buccal cortical bone reduction.

A 57-year-old asymptomatic male patient was referred to Istanbul Medipol University, Faculty of Dentistry, Department of Dentomaxillofacial Radiology in order to undergo routine periodontal treatment. Medical history and dental history were not contributory. Defect of the right mandibular corpus was detected during routine panoramic imaging. Palpation of the defect was not painful with no discomfort. Cone beam computerized tomography (CBCT) was found appropriate for further evaluation. Results showed an oval-shaped, radiolucent area of cystic aspect. This lithic area, located under the lower right second and third molar. The lower wall of the mandibular canal was visible within the radiolucent area showed that there could be a neighboring relationship, but not an involvement, of the inferior alveolar nerve (Figure 3). Lingual wall of the basal bone displayed involvement of the lesion and dimensions of the defect were 22.5 × 10 × 5.5 mm depth (mesiodistal length, inferosuperior height, and buccolingual depth).

## 2. Discussion

The Stafne bone defect was first described by Stafne in 1942 [1]. Since then, numerous cases have been reported [1–4, 7, 11, 12]. The exact pathogenesis is still obscure. Stafne suggested that the cavity could result from a failure of normal bone deposition in the region formerly occupied by cartilage [1, 5]. However, the most widely accepted view is that the cavities develop as a result of a localized pressure atrophy of the lingual surface of the mandible from the adjacent salivary gland [13].

Stafne bone defect has anterior and posterior variants. The posterior variant is the most known variant of the defect and is located between the mandibular angle and first mandibular molar tooth below the inferior dental canal [1–11, 14–17]. The defect in our first case was located on the second molar region and soft tissue content was connected with the mouth floor through the lingual cortex. For the second case, the defect was located on the edentulous third molar region.

Stafne bone defects were asymptomatic, with a predilection for men between 50 and 70 years [2, 5, 7, 8]. Distinctively, ın the first case, Stafne bone defect was described for 31-year-old asymptomatic male patient. And the prevalence in published series ranges from 0.10% to 0.48% [1–4, 7–14]. In our clinic, 7922 patients were diagnosed in 1 year and it can be assumed that the prevalence of Stafne bone defect is nearly 0.025%.

The diagnosis of this defect is incidental, since patients do not usually present clinical symptoms [9]. In the orthopantomograph, the technique which usually first identifies this entity, a radiolucent image with a well-defined sclerotic border is generally observed, situated at a posterior location of the mandible, below the inferior dental canal [16, 17].

Surgery is not necessary for the treatment of anterior or posterior Stafne bone defect. Surgical exploration or biopsy should be performed in atypical cases or other suspected lesions. The bone defects of two patients in this study were asymptomatic and any other bone lesions, such as cysts and tumors, were excluded because no signs of inflammatory or tumoral changes were evident [3]. Therefore, surgery was not considered and the patients were followed for 1 year. No remarkable changes of the defects were seen during the follow-up period; thus, justifying the accuracy of the initial diagnosis of posterior Stafne bone defect.

The differential diagnosis of SBC includes benign and malign jaw lesions such as odontogenic cystic lesion, nonossifying fibroma, fibrous displasia, vascular malformation, focal osteoporotic bone marrow defect, brown tumor of hyperparathyroidism, ameloblastoma, basal cell nevus syndrome, giant cell tumor, or a metastasis from a primary malignant tumor. Therefore, in some cases more confirmatory diagnostic tools are mandatory [2–5, 12, 18]. Sialography is able to depict salivary tissue in the bony cavity and has been used to confirm the diagnosis [19]. However, there were case reports of surgically proved Stafne bone cavity with negative results in sialography [20]. CT, currently considered as the complementary test of choice, has the great advantage of verifying the peripheral origin of the lesion and the conservation of the lingual cortical, which are essential characteristics for discounting other pathologies such as apical or residual cysts, fibrous dysplasia, and traumatic osseous cyst, among others [8, 17]. The fact that CT is more specific to bone lesions of the jaws and much less so to soft tissue have led some authors to advocate MR imaging as the primary diagnostic technique [6, 8]. Branstetter et al. [6] were the first to establish a diagnosis of SBC merely on MR imaging with no further treatment. The main advantage of MR imaging is its superior soft tissue characterization and discrimination. The superior soft tissue contrast of MR imaging should be adequate to make the diagnosis of SBC, even without any intravenous contrast material. Its major disadvantage is the high cost and the distortion artifacts produced by dental material [5]. In this paper, orthopantomograph and CBCT are used for diagnosing the defect.

## 3. Conclusion

Stafne bone defect was an incidental finding, presenting no evolutionary changes, and as such conservatory therapy based on periodic controls was indicated. Currently, complementary techniques such as CT are sufficient to establish a certain diagnosis.
